# Antitumor activity and inhibitory effects on cancer stem cell-like properties of Adeno-associated virus (AAV) -mediated Bmi-1 interference driven by Bmi-1 promoter for gastric cancer

**DOI:** 10.18632/oncotarget.8174

**Published:** 2016-03-18

**Authors:** Xiaowei Zhang, Weijian Guo, Xiaofeng Wang, Xinyang Liu, Mingzhu Huang, Lu Gan, Yufan Cheng, Jin Li

**Affiliations:** ^1^ Department of Medical Oncology, Fudan University Shanghai Cancer Center, Department of Oncology, Shanghai Medical College, Fudan University, Shanghai, China; ^2^ Shanghai Tianyou Hospital of Tongji University, Shanghai, China

**Keywords:** Bmi-1, RNA interference, gastric cancer, cancer stem cell

## Abstract

Bmi-1 is aberrantly activated in various cancers and plays a vital role in maintaining the self-renewal of stem cells. Our previous research revealed that Bmi-1 was overexpressed in gastric cancer (GC) and it's overexpression was an independent negative prognostic factor, suggesting it can be a therapeutic target. The main purpose of this investigation was to explore the antitumor activity of Bmi-1 interference driven by its own promoter (Ad-Bmi-1i) for GC. In this study, we used adenoviral vector to deliver Bmi-1 shRNA driven by its own promoter to treat GC. Our results revealed that Ad-Bmi-1i could selectively silence Bmi-1 in GC cells which overexpress Bmi-1 and suppress the malignant phenotypes and stem-like properties of GC cells *in vitro* and *in vivo*. Moreover, direct injection of Ad-Bmi-1i into xenografts suppressed tumor growth and destroyed cancer cells *in vivo*. Ad-Bmi-1i inhibited the proliferation of GC cells mainly via inducing senescence *in vitro*, but it suppressed tumor through inducing senescence and apoptosis, and inhibiting angiogenesis *in vivo*. Bmi-1 knockdown by Ad-Bmi-1i downregulated VEGF via inhibiting AKT activity. These results suggest that Ad-Bmi-1i not only inhibits tumor growth and stem cell-like phenotype by inducing cellular senescence directly, but also has an indirect anti-tumor activity by anti-angiogenesis effects via regulating PTEN/AKT/VEGF pathway. Transfer of gene interference guided by its own promoter by an adeno-associated virus (AAV) vector might be a potent antitumor approach for cancer therapy.

## INTRODUCTION

Gastric cancer is one of the most common malignancies and one of the leading causes of cancer-related death throughout the world. The prognosis of patients with metastatic gastric cancer is poor, with a median overall survival of 9–11 months with all available chemotherapy [1]. The use of targeted agents such as bevacizumab, cetuximab, lapatinib, and everolimus does not prolong overall survival significantly [2, 3]. Therefore, new treatment strategies are urgently needed for this ‘difficult to cure’ disease. Although it does not yet clearly explain the poor efficacies of chemotherapy and targeted therapy, evidence suggests that the existence of cancer stem cells (CSCs) in many cancers, including gastric cancer, is responsible for cancer initiation, metastasis, and treatment failure. Bmi-1 is one of the polycomb group of genes, which functions as an oncogene in many cancers [4]. In addition to its role in tumorigenesis and tumor progression, Bmi-1 also plays an important part in self-renewal of hematopoietic stem cells, neural stem cells, mammary stem cells [5–8], and CSCs in some cancers. Recently, we found that Bmi-1 was highly expressed in gastric cancer tissues, was correlated with depth of invasion and lymph node metastasis, and was an independent risk and prognostic factor associated with poor survival [22] Our recent research results also revealed that Bmi-1 might also play a vital role in maintaining the properties of gastric CSCs (Supplementary Figure 1). Therefore, Bmi-1 might be a good therapeutic target for CSCs.

Several studies have shown that Bmi-1 RNA interference (RNAi) can inhibit proliferation of cancer cells and increase chemosensitivity [9–12]. Our previous study showed that inhibition of Bmi-1 by its short hairpin RNA (shRNA) inhibited the malignant phenotypes in cancer cells. However, the difficulty of expressing RNAi system and silencing target gene specifically in cancer cells and tissues is the main barrier of RNAi technique for its' *in vivo* utility.

Studies have shown that AAV vector regulated by tissue- or cell-specific promoters, including carcinoembryonic antigen promoter and alpha-fetoprotein, can have a relatively specific tumor targeting effect [13–16].

In this study, we engineered a AAV vector to deliver Bmi-1 shRNA driven by its own promoter to treat gastric cancer *in vitro* and *in vivo*. Silencing Bmi-1 specifically in Bmi-1 highly-expressed gastric cancer cells was expected to achieve the inhibition of the malignant and stem cell-like phenotypes.

## RESULTS

### Construction of Bmi-1 shRNA AAV vector and its selective Bmi-1 interference role for different GC cells *in vitro*

**Figure 1:**
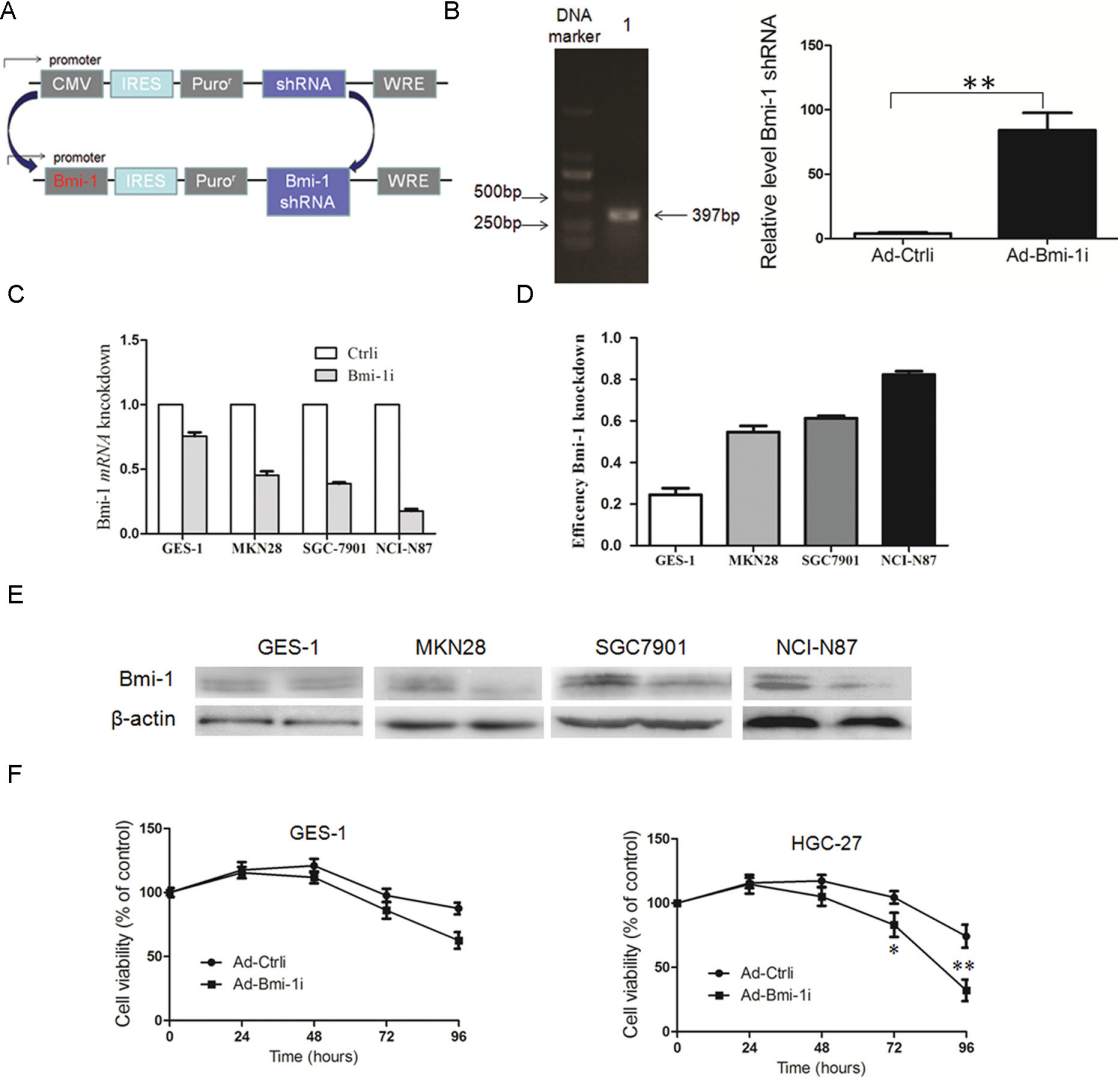
Construction of Ad-Bmi-1i vector (Bmi-1i) and its Bmi-1 interference efficacy for GC cells with different Bmi-1 expression levels *in vitro* (**A**) Schematic diagram of the recombinant adenovirus Ad-Bmi-1i. (**B**) PCR band of the Bmi-1 promoter. The total length of Bmi-1 promoter was 397 bp (DNA marker ladders:150 bp, 250 bp, 500 bp, 750 bp, 1000 bp, and 2000 bp) (*left panel*), Bmi-1 shRNA transcripts in control and Ad-Bmi-1i infected AGS cells detected by QRT-PCR (*right panel*). (**C**) Selective Bmi-1 silencing efficacy of Ad-Bmi-1i *in vitro*. Higher Bmi-1 silencing efficiency was found in SGC-7901 and NCI-N87 cells with relatively higher Bmi-1 expression than that in cancer cells MKN28 and immortal normal cell lines (GES-1) with lower Bmi-1 expression infected with Ad-Bmi-1i. The cells were collected after 48 hours of infection for analysis of Bmi-1 mRNA and protein by qRT-PCR and Western blot, respectively. (**D**) Induction of cytotoxicity induced by Ad-Bmi-1i *in vitro*. HGC-27 and GES-1 were infected with Ad-Bmi-1i (Bmi-1i) or Ad-Ctrli (Ctrli). Cell viability was determined by CCK8 assay at different times after transfection. *P < 0.05, **P < 0.01. (data are represented as mean ± SD).

In order to examine the anti-tumor effect of Bmi-1 shRNA for cancer cells, we engineered recombinant adenoviral vectors carrying Bmi-1 shRNA driven by Bmi-1 promoter (Ad-Bmi-1i) (Figure 1A and 1B). The core promoter sequence primer was synthesized by Invitrogen. The promoter activity of this sequence has been described previously [17]. Bmi-1 shRNA was designed and cloned in the adenoviral vector obtained from Shanghai Shengbo Biotechnology Company. The sequence of Bmi-1 shRNA was as follows: Bmi-1 shRNA GACCAGACCACUACUGAAU [17]. In order to prove that Bmi-1 shRNA could efficiently transcribed by Bmi-1 promoter, we designed a prober targeting Bmi-1 shRNA sequence, and deteceted the expression of Bmi-1 shRNA in control and Ad-Bmi-1i infected AGS cells detected by QRT-PCR (Figure 1B).

We evaluated the specifically silencing efficiency of Ad-Bmi-1i for gastric cancer *in vitro*. Firstly, we compared the Bmi-1 silencing efficiency for GES-1 (normal immortal gastric mucosal cells) and gastric cancer cells with different Bmi-1 expression levels. Results revealed that higher Bmi-1 interference rate was found by Ad-Bmi-1i in cancer cells with higher Bmi-1 expression, while lower Bmi-1 interference rate was found in GES-1 cells and gastric cancer cells with lower Bmi-1 expression, which means that the higher Bmi-1 silencing efficiency was obtained in gastric cancer cells with higher Bmi-1 expression (Figure 1C), suggesting that Ad-Bmi-1i selectively expressed and exerted silencing effects in cancer cells with higher Bmi-1 expression. Furtherly, we explored the cytotoxicity of Ad-Bmi-1i detected by Cell Counting Kit (CCK-8) assay, and showed that less toxicity was found for GES-1 cells compared with gastric cancer with higher Bmi-1 expression (Figure 1D).

### Knockdown of Bmi-1 by Ad-Bmi-1i reduces malignant phenotype and stem-like property of gastric cancer cells *in vitro*

**Figure 2:**
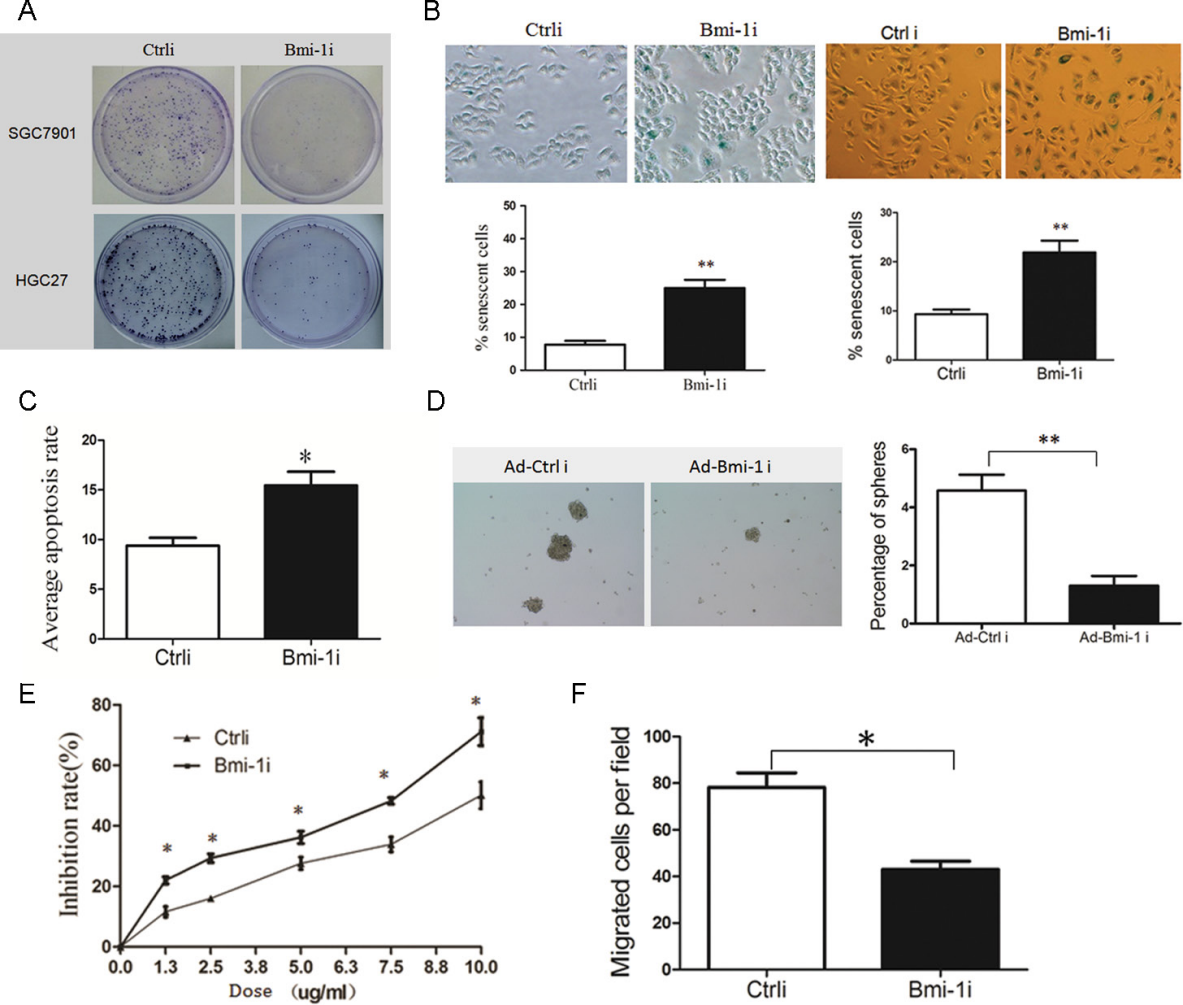
Knockdown of Bmi-1 by Ad-Bmi-1i transfection decreases malignant phenotypes in GC cells *in vitro* (**A**) Knockdown of Bmi-1 decreases proliferative ability detected by plate colony formation assay in stably Bmi-1 knockdown SGC-7901 and HGC-27 cells. (**B**) Knockdown of Bmi-1 results in induction of cellular senescence. Cellular senescence was stained using SA-β-gal assay in SGC-7901 (*left*) and HGC-27 (*right*) cells, stable Bmi-1 knockdown (Ad-Bmi-1i) or control vector (Ad-Ctrli) (upper), and quantified (lower). (**C**) Knockdown of Bmi-1 in SGC-7901 results in slightly increase of cellular apoptosis *in vitro* detected by the Annexin V-propidium iodide apoptosis detection. (**D**) Bmi-1 inhibition induced by Ad-Bmi-1i reduced gastric CSC self-renewal activity *in vitro*. Bmi-1 knockdown cells and its control cells were cultured using serum-free microsphere culture method. (**E**) Bmi-1 knockdown sensitizes gastric cancer cells to chemotherapy in SGC-7901 cells. Bmi-1 knockdown cells and its control cells were treated with 5-fluorouracil detected by CCK8 after 48 hours. For a dose-dependent assay, the cells were administered with different doses of 5-fluorouracil, and cell survival was determined 48 hours after administration. (**F**) Bmi-1 knockdown inhibits migration in SGC-7901 cells. Transwell migration assay was performed using the Corning Transwell chamber (8 μm), pictures of migrated cells was showed in *left panel*, and quantitation of migrated cells in *right panel*. (data are represented as mean ± SD) *P < 0.05, **P < 0.01.

To analyze the anti-tumor efficiency of Ad-Bmi-1i, gastric cancer cells and GES-1 were infected with Ad-Bmi-1i, and cells proliferative ability was determined by plate colony formation assay. Ad-Bmi-1i suppressed the proliferative ability of gastric cancer cells (Figure 2A), but had little effect on GES-1 cells (Data not shown).

Cellular senescence constitutes a powerful barrier to carcinogenesis [18, 19], and our previous studies showed that knockdown of Bmi-1 by Bmi-1 shRNA can induce cellular senescence in gastric cancer cells. In this study, we also detected senescence by SA-β-gal staining and found that Ad-Bmi-1i significantly induced cellular senescence (Figure 2B). Furthermore, we observed slightly increased cell apoptosis in Ad-Bmi-1i infected cells detected by Annexin V-PI (propidium iodide) staining compared with that in control cells(infected by Ad-Ctrli) (Figure 2C).

As Bmi-1 is one of the stem cells markers and plays an important role in maintaining self-renewal of stem cells and some kinds of CSCs, it may also be a good target of gastric CSCs. Firstly, we check the influence of Bmi-1 on gastric stem cell-like properties. Our previous research has revealed that isolated spheroid cells from GC cell lines and primary cancer cells by serum-free culture method have stem cell-like properties, suggesting microsphere enriches CSCs or stem-cell-like cells [20]. So we used serum-free culture microsphere formation to measure the self-renewal ability of stem-like cells, and our results revealed that Bmi-1 overexpression promotes the self-renewal ability of gastric cancer cells. Furthermore, we also found that Bmi-1 overexpression increased migration ability and drug resistance in gastric cancer cells *in vitro*, which are also characteristics of CSCs (Supplementary Figure 1).

Secondly, we investigated the effects of Ad-Bmi-1i on stem cell-like properties. Spheroid Colony-formation Assay revealed that Bmi-1 inhibition induced by Ad-Bmi-1i suppresses the self-renewal activity in SGC-7901 GC cells (Figure 2D). CSCs are thought to sustain metastasis and chemoresistance [19], and we also found that Bmi-1 knockdown by Ad-Bmi-1i sensitized gastric cancer cells to chemotherapy (Figure 2E) and deceased cell migration ability (Figure 2F). These data indicate that Ad-Bmi-1i can suppress the malignant phenotypes and stem cell-like properties of GC cells *in vitro*.

### Bmi-1 interference by Ad-Bmi-1i suppresses GC xenografts in nude mice models by induction of cellular senescence, promotion of apoptosis, or suppression of angiogenesis

**Figure 3:**
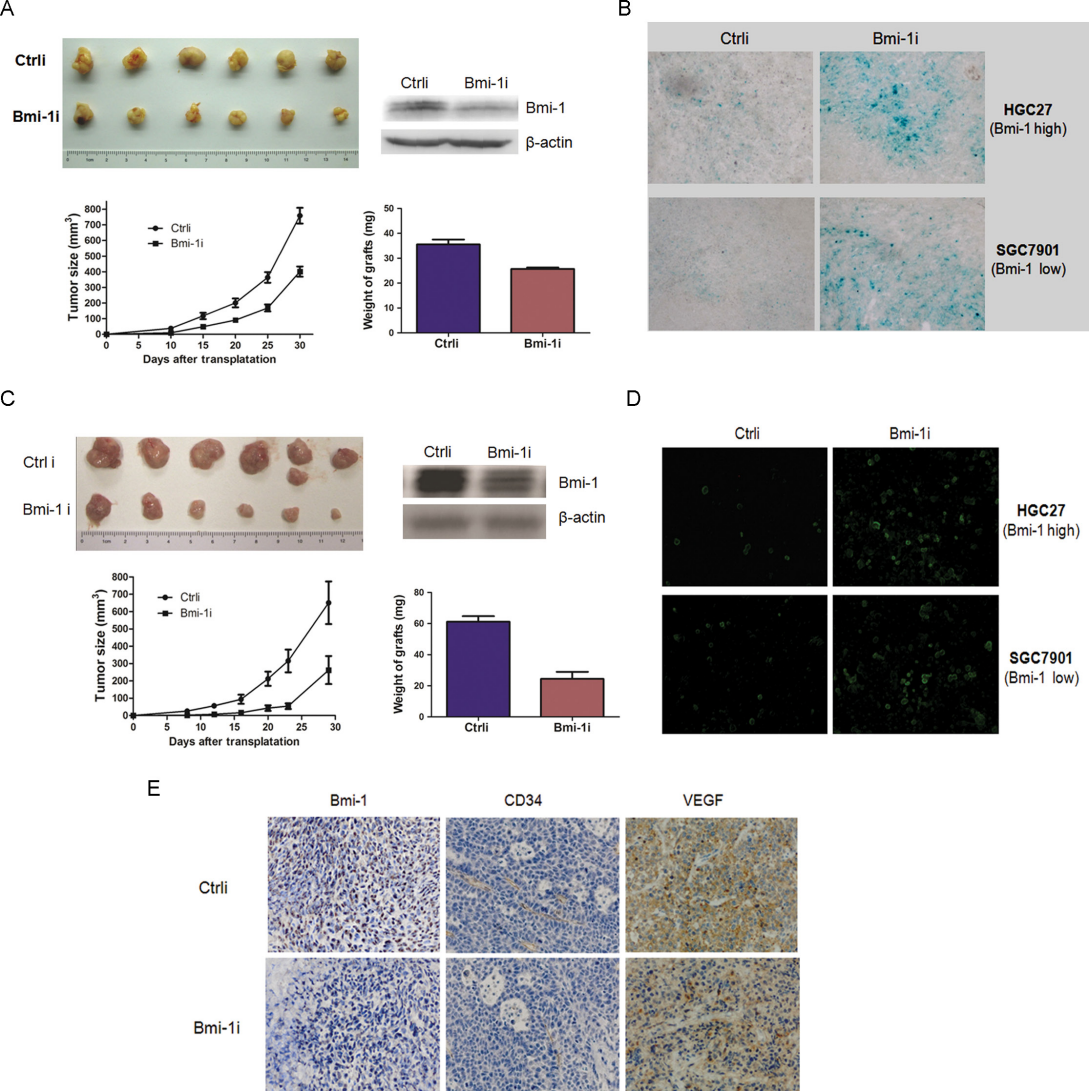
Ad-Bmi-1i suppresses tumor growth in GC xenografts *in vivo* (**A**) Ad-Bmi-1i suppresses tumor growth in SGC-7901 GC cells. Growth curves of xenografts after subcutaneous injection of control (Ctrli) and stable Bmi-1 silencing cells by transfection of Ad-Bmi-1i in Balb/C mice (Bmi-1i). Data represent mean ± standard deviation (SD) (*n* = 6); the average weight of stable Bmi-1 silencing and control xenografts of SGC-7901 (*n* = 6) are represented as mean ± SD. (**B**) Ad-Bmi-1i suppresses tumor growth in HGC-27 GC cells. Growth curves of tumors after subcutaneous injection of control and stable Bmi-1 silencing cells by transfection of Ad-Bmi-1i in Balb/C mice. Data represent mean ± SD (*n* = 6); the average weight of stable Bmi-1 silencing and control xenografts of SGC-7901 (*n* = 6) are represented as mean ± SD. (**C**) Representative images of *in vivo* senescence staining show the grafts and microscopic phenotypes of stable Bmi-1 interference or control tumors (SGC-7901 and HGC-27). SA-β-gal (blue) staining of representative sections; bars = 100 μm. (**D**) Representative images of *in vivo* cell apoptosis show the grafts and microscopic phenotypes of stable Bmi-1 interference or control tumors (SGC-7901 and HGC-27). TUNEL (green) staining of representative sections; bars = 200 μm. (**E**) Expression levels of CD34 (microvessel density) and VEGF were decreased in Bmi-1 knockdown cells, detected by IHC. *P < 0.05, **P < 0.01.

To analyze the anti-tumor efficiency of Ad-Bmi-1i *in vivo*, the control and stable Bmi-1-silencing SGC-7901 cells infected by Ad-Bmi-1i (5*10^6^) were injected subcutaneously into one rear flank of BALB/c mice and tumor growth was examined. Mice injected with stable Bmi-1-silencing cells formed smaller tumors within 24 days than those injected with control cells (Figure 3A and 3B). The inhibitory efficiency for HGC-27 cells with higher Bmi-1 expression was higher than that for SGC-7901 cells with lower Bmi-1 expression, suggesting that Ad-Bmi-1i might have a specific inhibitory effect for gastric cancer *in vivo*.

The induction of cellular senescence by Ad-Bmi-1i in tumor tissues was examined *in vivo*. Isolated frozen xenograft slices were stained for SA-β-gal marker. The results showed a significantly higher percentage of senescent cells present in the Ad-Bmi-1i group (Figure 3C). Examination of the isolated xenograft slices for cellular apoptosis *in vivo* via TUNEL staining showed that a significantly higher percentage of apoptotic cells were present in the Ad-Bmi-1i group, which was different from the induction of cellular apoptosis by Bmi-1 interference *in vitro*. Similar results were also found for HGC-27 cancer cells, suggesting that Bmi-1 interference by Ad-Bmi-1i infection also inhibits tumor growth by inducing both cellular senescence and apoptosis *in vivo* (Figure 3C).

We also investigated the *in vivo* role of Bmi-1 interference for angiogenesis by using the HGC-27 xenograft mouse model, and immunohistochemical assay was employed to show the microvessels detected by CD34, and VEGF expression, which is involved in angiogenesis [21]. The results showed that Bmi-1 silencing xenografts have a lower density of microvessels and lower expression of VEGF (Figure 3D), suggesting that Bmi-1 silencing might inhibit tumor angiogenesis via downregulation of VEGF. These results suggest that Ad-Bmi-1i may have an indirect anti-tumor role by anti-angiogenesis.

### Anti-tumor activity by Ad-Bmi-1i injection in an animal model with subcutaneous xenografts

**Figure 4:**
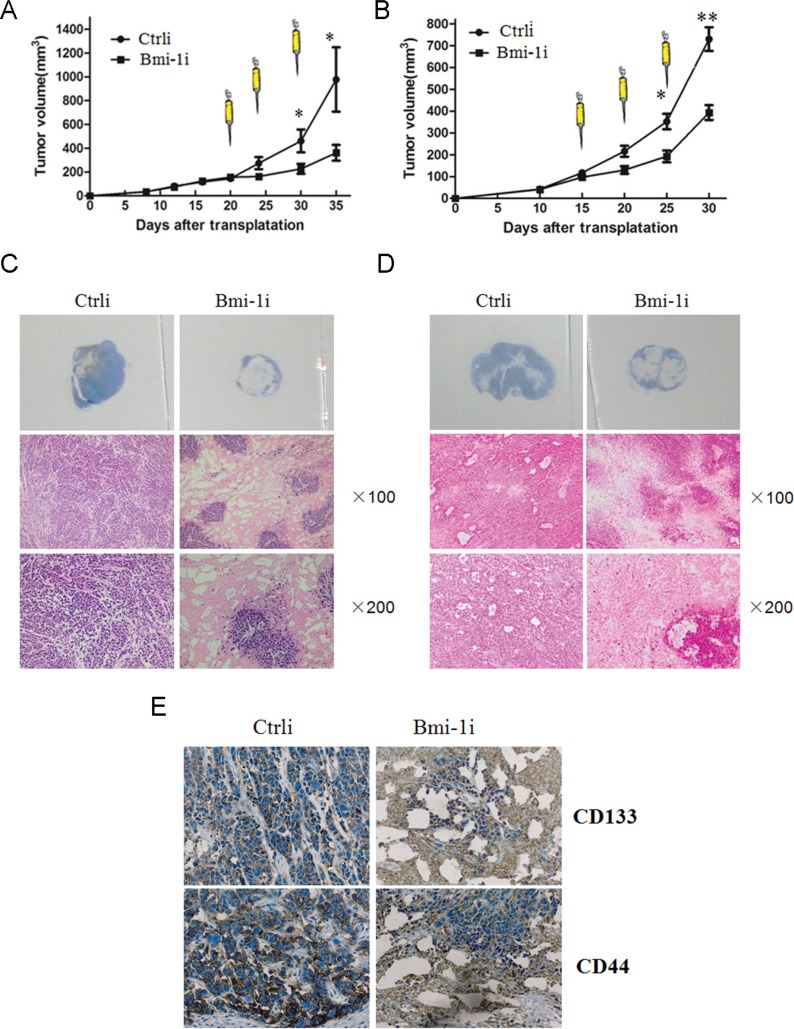
Effect of Ad-Bmi-1i on established GC xenografts After reaching a size of 4 mm in diameter, tumors were treated with three injections (arrows) of control vector (Ctrli) or Ad-Bmi-1i (Bmi-1i) (total dose 3 × 10^8^ pfu) on the indicated days. Tumor volume was plotted for each treatment animal group (*n* = 6) against days after inoculation (mean ± SD). (**A**) Over the 35-day experimental period, SGC-7901 xenografts were significantly suppressed in Ad-Bmi-1i treated group in comparison with those in control group (*P* < .05) (left panel). Over the 30-day experimental period, HGC-27 xenografts were significantly suppressed in Ad-Bmi-1i treated group in comparison with those in control group (*P* < .05) (right panel). B. Hematoxylin and eosin (H & E) staining showed that the cancer cells in the control group grew well, with only small patches of necrosis, and cells in Ad-Bmi-1i group had large patches of necrosis in SGC-7901 xenografts (**B**) and HGC-27 xenografts (**C**). (**D**) The possible killing or inhibitory role of Ad-Bmi-1i for CSC-like cells *in vivo*: IHC was used to detect expression of CD133 and CD44 in the HGC-27 cell xenografts treated with Ad-Bmi-1i or control vector.

To assess the efficacy of Ad-Bmi-1i treatment for subcutaneous xenografts, SGC-7901 (lower Bmi-1 expression) and HGC-27 (higher Bmi-1 expression) human GC xenograft models were established in nude mice. When the xenograft s grew to 180–220 mm^3^, recombinant AAV vector (Ad-Bmi-1i) or a control vector (Ad-Ctrli) was injected directly into the subcutaneous xenografts. The growth of SGC-7901 xenografts was significantly inhibited by direct injection of Ad-Bmi-1i, compared with treatment with a control vector (*P* < .001) (Figure 4A *left panel*). Average tumor volumes on day 30 were 43 mm^3^ for the Ad-Bmi-1i treated mice and 332 mm^3^ for those treated with Ad-Ctrli. more significant inhibition rate was found in HGC-27 cells (Figure 4A *right panel*) compared with that in SGC-7901 cells. Data from these animal experiments showed that Ad-Bmi-1i injection could suppress the growth of established tumors derived from gastric cancer cells.

Furtherly, we detected the inner change of gastric cancer xenografts. Hematoxylin and eosin staining showed that cancer cells in the control group grew well, with only small patches of necrosis, while cells in the Ad-Bmi-1i treated group had more patches of necrosis area (Figure 4B and 4C), suggesting the direct killing effect of Ad-Bmi-1 is present.

### Potential inhibitory role of Ad-Bmi-1i for gastric cancer stem-like cells *in vivo*

Our previous results have found that CD133-positive and CD44-positive cells have the properties of gastric CSCs [20]. To determine whether Ad-Bmi-1i has inhibitory or killing effect on gastric CSC-like cells, we detected the expression of CD133 and CD44 by Immunohistochemistry (IHC) in Ad-Bmi-1i treated gastric cancer xenografts (Ad-Bmi-1i) and in control group (Ad-Ctrli). There was less percentage of CD133-positive and CD44-positive cancer cells in the Ad-Bmi-1i-treated xenograft tissues than in control group (Figure 4D), suggesting a potential inhibitory or killing role of Ad-Bmi-1i for gastric CSCs.

### Bmi-1 upregulates AKT activity, and VEGF pathways in gastric cancer cell lines

**Figure 5:**
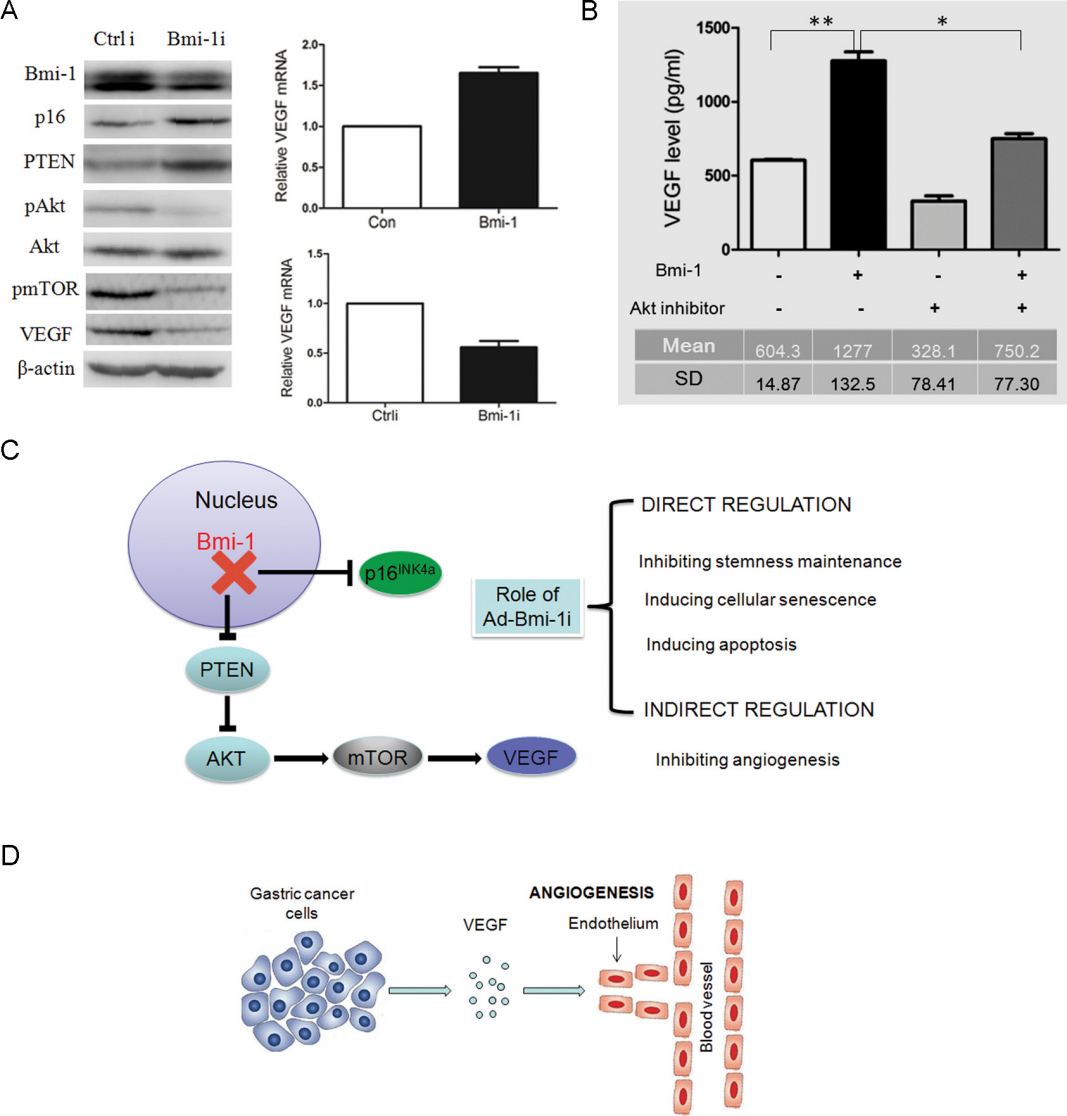
Knockdown of Bmi-1 by Ad-Bmi-1i transfection results in the downregulation of VEGF via the PTEN/AKT/mTOR pathway (**A**) Knockdown of Bmi-1 leads to upregulation of PTEN proteins, and reduction in pAKT (phosphorylated AKT), downregulation of p-mTOR and downregulation of VEGF expression as determined by Western blot analysis. β-actin is an internal control (*left panel*). Overexpression of Bmi-1 leads to increase of VEGF mRNA expression as determined by qRT-PCR analysis; glyceraldehyde 3-phosphate dehydrogenase (GAPDH) is an internal control (*upper right panel*). Transient transfection of Bmi-1 short hairpin RNA (shRNA) in MKN45 cells results in decrease of VEGF mRNA expression as determined by qRT-PCR. GAPDH is an internal control (*upper right panel*). (**B**) AKT inhibitor (cell signaling Wortmannin #9951, 0.25 uM) can partially reverse the increased VEGF secretion induced by Bmi-1 overexpression in gastric cancers. The VEGF concentrations in fixed 2 ml supernatant were detected by ELISA assay in SGC-7901 cells expressing Bmi-1 or Bmi-1 together with AKT inhibitor (mean ± SD). (**C**) The two molecular pathways (p16 and PTEN/AKT/mTOR/VEGR) of Bmi-1 shRNA in GC. Ad-Bmi-1i induces upregulation of p16 expression, eventually leading to the direct killing or inhibitory role (blockage of stemness maintenance, inducing cellular senescence or apoptosis) and indirect killing or inhibitory role (decreasing formation of blood vessels surrounding the tumor) for gastric cancer. (**D**) The regulation of Bmi-1 for angiogenesis in gastric cancer cells. Bmi-1 overexpression can increase the secretion of VEGF in SGC-7901 cells, and subsequently promote angiogenesis surrounding the tumor, further inducing the progression of cancer via the indirect or direct role. *p < 0.05, **p < 0.01.

To determine the possible molecular mechanisms of the anti-tumor and inhibition of blood vessels role of Ad-Bmi-1i treatment, we examined the expression of p16 and p-AKT in control and Bmi-1 knockdown cells. Consistent with our previous data [22] we found that Ad-Bmi-1i can downregulate phosphorylated-AKT (pAKT). Besides this, Bmi-1 downregulation also leads to downregulation of pmTOR and reduction of VEGF in SGC-7901 cells (Figure 5A). Combined with the already known PTEN/AKT/mTOR pathway [23, 24], we assumed that Bmi-1 might upregulate VEGF expression via the PTEN/AKT/mTOR pathway. To clarify this, firstly, we confirmed that Bmi-1 overexpression increased the expression of VEGF mRNA detected by qRT-PCR and Bmi-1 knockdown downregulated VEGF mRNA expression (Figure 5A). Furtherly, we inhibited AKT activity using an AKT inhibitor in Bmi-1-overexpressing cells by transfection of Bmi-1 overexpressing plasmid, and found that AKT inhibitor could reverse Bmi-1-mediated upregulation of VEGF secretion detected by ELISA (enzyme-linked immunosorbent assay) using the cell culture supernatant (Figure 5B), revealing AKT to be a mediator of Bmi-1 regulating VEGF.

## DISCUSSION

Technological advancements in the molecular characterization of cancers have enabled researchers to identify an increasing number of key molecular drivers of cancer progression, which have led to multiple novel anticancer therapeutics, and clinical benefit in selected patient populations [25]. Aberrant expression of Bmi-1 has been found in several human cancers and its overexpression is often correlated with poor prognosis in many types of cancers. Most importantly, Bmi-1 is required for self-renewal of stem cells and some kinds of CSCs [5, 26, 27]. Hence, Bmi-1 is considered an important therapy target [5, 28–31]. Our previous findings also showed that Bmi-1 acts as an oncogene in gastric cancer and an independent prognostic factor in gastric cancer tissues [32], suggesting it can also be a good therapeutic target for gastric cancer. Bmi-1 was found to be one of the CSCs drivers based on these findings.

Concerning gene therapy for cancer, the ideal gene delivery vectors is that can safely, efficiently, and specifically deliver genetic material to the target cells [33]. Initial efforts to deactivate oncogenes and replace non-functioning tumor suppressor genes were presently commercially available. In the past decade, many genes, including Canstatin [34], VEGF [35], drug resistance gene MDR1 [36, 37], stem cell marker CD44 [38], and tumor suppressor p53 [39] have been constructed into vectors for investigation of anti-tumor effects.

Adeno-associated virus, which represents small, single-stranded DNA viruses, which do not usually cause infection without co-infection of a helper virus [33]. AAV vectors have enjoyed increasing clinical success as a result of their excellent gene delivery efficacy, lack of pathogenicity, strong safety profile and gene expression in post-mitotic cells [33, 40, 41]. These excellent gene delivery properties have been harnessed for *in vitro* cancer studies, *in vivo* pre-clinical cancer models, and more recently cancer clinical trials under development [33], but few are presently available. ONYX-015 (Onyx Pharmaceuticals) is a modified oncolytic adenovirus in the treatment of refractory head and neck cancer combined with cisplatinum, which had been approved by the Chinese Food and Drug Administration in 2005 [42]. The limited success has been achieved mainly due to relatively high toxicity, low transfection efficiency, the risk of mutagenesis and second malignancies induced by genetic instability of carried genes [33, 43], and a limited potential for sustained anti-tumor effects [40].

Gene targeted siRNA is not only a method for gene function study, but also a potential therapeutic method. AAV encoding small hairpin RNA (shRNA) targeting LMP-1 [44] and FHL2 [45] have been well employed in a variety of tumor models. Previously we have already found that Bmi-1 knockdown by shRNA inhibited the proliferation and induced senescence of GC cells. However, as normal cells also lowly express Bmi-1, Bmi-1 siRNA may also inhibit and kill normal cells *in vivo*. So how to express siRNA selectively in cancer cells is the main problem.

Tumor cell-specific promoters are usually used to increase the specific expression of the target gene in cancer cells. Carcinoembryonic antigen promoter, mucin-like glycoprotein episialin promoter, alpha-fetoprotein promoter, Tyr promoter, and Epstein-Barr virus promoter were reported to have enhanced activities [46–50]. However, all these already-reported gene targeted vectors are controlled by different gene promoters, which could affect the vectors' efficiency if there is difference of expression level between the target gene and the promoter gene in the targeted cells. Here, in this study we constructed an AAV vector (Ad-Bmi-1i) containing Bmi-1 shRNA driven by its own promoter, which could specifically express and function in cancer cells with higher Bmi-1 expression. Our results revealed that Ad-Bmi-1i selectively silenced Bmi-1 expression in Bmi-1 relatively high-expressed cells, and Bmi-1 silence by Ad-Bmi-1i infection not only induced senescence and inhibited proliferation of GC cells *in vitro* and *in vivo*, but also decreased stem-like properties of GC cells. To evaluate the antitumor activity of Ad-Bmi-1i *in vivo*, we established an animal model using direct injection of Ad-Bmi-1i. Treatment with Ad-Bmi-1i resulted in significant tumor regression, and pathologic analysis showed that most of the cancer cells were destroyed. Further IHC analysis showed that the percentages of remaining alive CD133- or CD44-positive cells (reported markers of gastric CSCs [51–53]) was significantly reduced in Ad-Bmi-1i injected group, compared with in control group. The results implied that Ad-Bmi-1i could inhibit the amplifying cancer cells as well as gastric CSCs. As CSCs are a unique subpopulation of cells that possess self-renewal and differentiation potential and are responsible for tumor initiation, invasion, metastasis, and treatment failure, a therapeutic modality targeting CSCs might lead to eradication of a tumor.

Furtherly we investigated the mechanisms of Ad-Bmi-1i in treating GC. Consistent with previous data [32], we found that Ad-Bmi-1i upregulated p16, and downregulated phosphorylated-AKT (pAKT), which are the already known mechanisms of Bmi-1 knockdown in inducing senescence and inhibiting GC cells directly. Interestingly, our *in vivo* xenograft mice model experiments revealed that microvessel density (MVD) was obviously reduced in Ad-Bmi-1i group (Figure 3D), and Ad-Bmi-1i inhibited the proliferation of GC cells mainly via inducing senescence *in vitro* but induced both senescence and apoptosis *in vivo* (slight increase of apoptosis was found in *in vitro* but a more obvious increase of apoptosis was found *in vivo* in Ad-Bmi-1i group), suggesting that Ad-Bmi-1i could not only reduce malignant phenotypes directly, but also inhibit tumor growth indirectly by anti-angiogenesis effects which induced apoptosis *in vivo*. It's a novel finding that Bmi-1 involves in tumor angiogenesis, and what's the mechanism?

VEGF is well known as a pivotal inducer of tumor angiogenesis and metastasis [54, 55]. A number of studies have demonstrated that PI3K/PTEN/AKT/mTOR signaling pathway regulates VEGF expression in different types of cancer cells [56, 57], and it was found in previous and present studies that Bmi-1 upregulates AKT activity, so we speculated that Bmi-1 might upregulate VEGF expression via activating AKT, and VEGF might mediate the function of Bmi-1 in inducing angiogenesis. Actually our results showed that Bmi-1 knockdown by Ad-Bmi-1i treatment upregulated PTEN, and downregulated pAKT, p-mTOR, and VEGF protein detected by Western *in vitro* and also decreased VEGF protein expression *in vivo* detected by IHC, and Bmi-1 overexpression increased the expression of VEGF mRNA and Bmi-1 knockdown downregulated VEGF mRNA expression level, which confirmed that Bmi-1 upregulates VEGF expression. Furtherly, AKT inhibitor treatment could reverse Bmi-1-mediated upregulation of VEGF secretion in cell culture supernatant, which confirmed that Bmi-1 upregulates VEGF via activating AKT.

Taken together, Ad-Bmi-1i selectively silence Bmi-1 expression in Bmi-1 overexpressed GC cells, and Bmi-1 knockdown by Ad-Bmi-1i not only inhibits tumor growth and stem cell-like phenotype by inducing cellular senescence directly, but also has an indirect anti-tumor activity by anti-angiogenesis effects via regulating PTEN/AKT/mTOR/VEGF pathway. Transfer of gene interference guided by its own promoter by an AAV vector might be a potent target antitumor approach for cancer therapy.

## MATERIALS AND METHODS

### Cellular reagents and methods

An immortalized human gastric mucosal epithelial cell line (GES-1) and human gastric cancer cell lines (MKN28, SGC-7901, SNU-1 and SNU-16) were obtained from the Surgical Institution of Ruijin Hospital, Shanghai, China. Human gastric cancer cell lines (MKN45, HGC-27, NCI-N87 and AGS), and other cancer cell lines were purchased from the Cell Resource Center, Shanghai Institute of Biochemistry and Cell Biology at the Chinese Academy of Sciences. These cell lines were cultured in RPMI-1640 or DMEM base medium supplemented with 10% fetal bovine serum (FBS) and antibiotics. For plate colony formation assay, cells were plated in a six-well plate (10^3^ cells/well) and incubated at 37°C for 14 days. After two washes in PBS, cells were stained with 0.5% crystal violet solution and images were obtained. The crystal violet was washed away with 33% acetic acid. For plate colony formation assay, cells were plated in a six-well plate (1*10^3^ cells/well) and incubated at 37°C for 14 days. After two washes in phosphate-buffered saline (PBS), cells were stained with 0.5% crystal violet solution and images were obtained. The crystal violet solution was washed away with 33% acetic acid. Senescence-associated β-galactosidase (SA-β-gal) assay was performed as described previously [58]. Cell apoptosis assay was performed as described previously [58]. Lentiviral vectors for Bmi-1 shRNA were used as described previously [59]. Stable cell lines overexpressing Bmi-1 gene was generated by infection of the retroviral vectors expressing the particular gene as described [59].

### Cell migration assay

Cell axsmigration ability was analyzed by the Transwell chamber assay. 10% FBS was used as a chemoattractant. Cells on the lower surface of the insert were fixed and stained followed by counting under a light microscope [60].

### Chemo-sensitivity experiment

Spheroid cells were inoculated into 96-well plates (2000 cells per well) in triplicate on the day prior to testing. Each well was supplied with RPMI-1640 medium containing 10% FBS, along with a chemotherapy reagent such as 2.5 ug/ml or 5 ug/ml 5-fluorouracil (5-FU) (Sigma-Aldrich), 0.25 ug/ml epirubicin (EPI) (Sigma-Aldrich) and no drug(DMSO) as control. The appropriate medium for each well was changed after 24 hrs from initial treatment. The number of viable cells was evaluated after 48 hours from initial treatment using the Cell Counting Kit-8 (CCK8) (Dojindo, Rockville, MD, http://www.dojindo.com) following the manufacturer's instructions, and the optical absorbance at wavelength 450 nm was measured for the supernatant of each well using the plate reader Multiskan EX (Thermo Fisher ScientificInc., Waltham, MA; http://www.thermofisher.com).

### Spheroid colony-formation assay

Spheroid Colony Formation Assay was carried out as described previously [51]. Human gastric cancer cells were inoculated in each well (10 cells per well or otherwise indicated) of ultra-low-attachment 96-well plates (Corning Life Sciences, Acton, MA, http://www.corning.com/lifesciences) supplemented with 100–200 μl of RPMI-1640

medium (Invitrogen) plus 10 mM HEPES, human recombinant epidermal growth factor (EGF) (Invitrogen) at the concentration of 20 ng/ml, and human recombinant basic fibroblast growth factor (bFGF) (Invitrogen) at the concentration of 10 ng/ml. After about 3 weeks, each well was examined using light microscope and total well numbers with spheroid colonies were counted.

### Antibodies and western blot analysis

Antibodies against Bmi-1 (#5856), PTEN (#9559), phospho-Akt (ser-473, #4060), Akt (#2920), CD34 (#3569), CD44 (#3570) and p-mTOR (#5536s) were obtained from Cell Signaling (Danvers, MA). Antibody against VEGF (ab46154) was purchased from Abcam (Cambridge, MA). Antibody against CD133(CD133/1 (AC133) pure 130-090-422) were purchased from Miltenyi Biotec (German). Anti-β-actin (Santa Cruz, CA) was used at a 1:5000 dilution. The whole cell lysates were harvested using cell lysis buffer supplemented with a protease inhibitor cocktail (Sigma), and Western blot analyses were performed as described previously [61].

### *In vivo* tumorigenesis, SA-Δ-Gal assay, TUNEL assay and IHC of frozen tissue sections

All animal experiments were performed with the approval of Fudan University Animal Care and Use Committee. Stabley-Bmi-1 knockdown SGC-7901 or HGC-27 and its control cells (5 × 10^6^ each) were injected s.c. in one rear flank of 4- to 5-week-old female severe combined immunodeficient (SCID) mice. The growth of primary tumors was monitored at every 4 or 5 day interval by measuring the tumor diameters. Tumor length (L) and width (W) were measured and tumor volume was calculated by the equation: volume = (W^2^ × L)/2. Mice were sacrificed 24 days after injection of the cells. Xenografts from SCID mice undergoing Moh's micrographic surgery were rapidly frozen in liquid nitrogen, and mounted in OCT. Thin sections (5 μm) were cut, mounted onto glass slides, fixed in 1% formalin in PBS for 1 min at room temperature, washed in PBS, immersed overnight in SA-Δ-Gal staining solution according to the manufacturer's instructions (Genmed GMS10012.3, Shanghai, China), and viewed under bright field at 100×magnification. IHC was performed by using a highly sensitive streptavidin-biotin-peroxidase detection system as described. All slides were interpreted by two independent observers in a blinded fashion. When more than 10% of the cells in a sample were stained with moderate to strong staining, the sample was considered positive. Otherwise, the samples were considered negative. TUNEL staining was performed using a Roche *In Situ* Cell Death Detection Kit (Cat. 11684817910) as described.

### Intra-tumor injection of Ad-Bmi-1i

SGC-7901 or HGC-27 and their control cells (5*10^6^ each) were injected subcutaneously into one rear flank of 4- to 5-week-old female severe combined immunodeficient deficiency (SCID) mice to establish the xeno-transplant tumors [62, 63]. After the tumors reached a size suitable for AAV vector injection at day 30, adenoviral-shRNA (Ad-Bmi-1i, 2.0 × 10^7^ particles/10 μL), PBS (10 μL), or adenoviral vector control (Ad-Ctrli, 2.0 × 10^7^ particles/10 μL) were injected into the tumors. The injection was divided to be given three or four times a day at day 4 or 5. The mice were sacrificed after 30 days and the tumors were isolated and weighed.

### Recombinant AAV vectors

The sequence of Bmi-1 promoter was synthesized by Invitrogen, The promoter activity of this Bmi-1 promoter has been verified in an earlier study [17]. Bmi-1 shRNAs were as follows: Bmi-1 shRNA GACCAGACCACUACUGAA, which has been verified previously [32]. Where relevant, cells were transfected with a control shRNA that does not match any known human or mouse coding cDNA, the availability of which has been verified in earlier research [59].

AAV vector Ad-Bmi-1i was constructed by a homologous recombination between the shuttle plasmid pAVsiRNA1.1 (Shanghai Shengbo Biotechnology Company, Shanghai, China) and the packing plasmid pBHGlox(delta)E1,3Cre (Shanghai Shengbo Biotechnology Company). The AAV promoter of pAVsiRNA1.1 was replaced by Bmi-1 promoter, and Bmi-1 shRNA was inserted. AAV vectors were isolated from a single plaque and expanded in 293 cells, after which the resultant viral solutions were purified and stored at −80°C. The virus titer was determined by plaque assay on 293 cells.

### Sequence of Bmi-1 promoter as follows

-400 AGAAAGAACGGGGGGCGGGGTGGGC TGCGCGGCGTGCGGGGCCGGAGCGGCGGCCGC GCGGAGCAGGGCGGCGCGTGTGGCGCTGTGGAG AAATGTCTCCGC CGCCGCGGCGCGGAGCGAGG GAGGGGCCGGGCGCGCGGCGCGGAGGGGAGGG GGCGGCCACGGGCCTGACTACACCGACACTAAT TCCCAGGCCGCCCTTA AGGAATGAGGGGAGCACG TGACCCGCTGGGGGGGCGGCGGGGGGAGGGG GCGGCGGACTCCGAGCCATTTTGGAGCCGGTGT CAGTTTCCACTCTGCCTTCAGCGGTGCATTTTTT TCCACCCTCCCCTCCCCCTCCTCCCCTCCCCCCG CTCGCACGCACACACACGGCGCCCCCCGCCCC CGCCTCCCCCA-1 mRNA start

### VEGF quantitation from cell culture supernatant

Production of VEGF from SGC-7901 cells was determined by enzyme-linked immunosorbent assay (ELISA) using ELISA kit (R & D systems, Minneapolis, MN). Cell culture supernatants were collected and assayed according to the manufacturers instructions. Cells was transfected with Bmi-1 overexpression, and Akt inhibitor (25 uM) Elisa was applied to detect the VEGF level secreted by gastric cancer cells.

### Statistical analysis

All statistical analyses were done by using the Statistical Package for the Social Sciences version 16.0 software (SPSS Inc, Chicago, IL, USA). A two-tailed *P* value less than 0.05 was considered statistically significant. In *in vitro* and *in vivo* experiments, data were described as mean ± standard deviation, and analyzed by student *t*-test.

## SUPPLEMENTARY MATERIALS FIGURE


