# Supplementary material for: Antitumor activity and inhibitory effects on cancer stem cell-like properties of Adeno-associated virus (AAV) -mediated Bmi-1 interference driven by Bmi-1 promoter for gastric cancer
Source: Oncotarget. 2016 Mar 18;7(16):22733–45. doi: 10.18632/oncotarget.8174 (PMC5008396; doi:10.18632/oncotarget.8174)
Supplement: Supplementary file 1 [file oncotarget-07-22733-s001.pdf]

Antitumor activity and inhibitory effects on cancer stem cells-like properties of Adeno-associated virus (AAV) -mediated Bmi-1 interference driven by Bmi-1 promoter for gastric cancer

Supplementary Materials

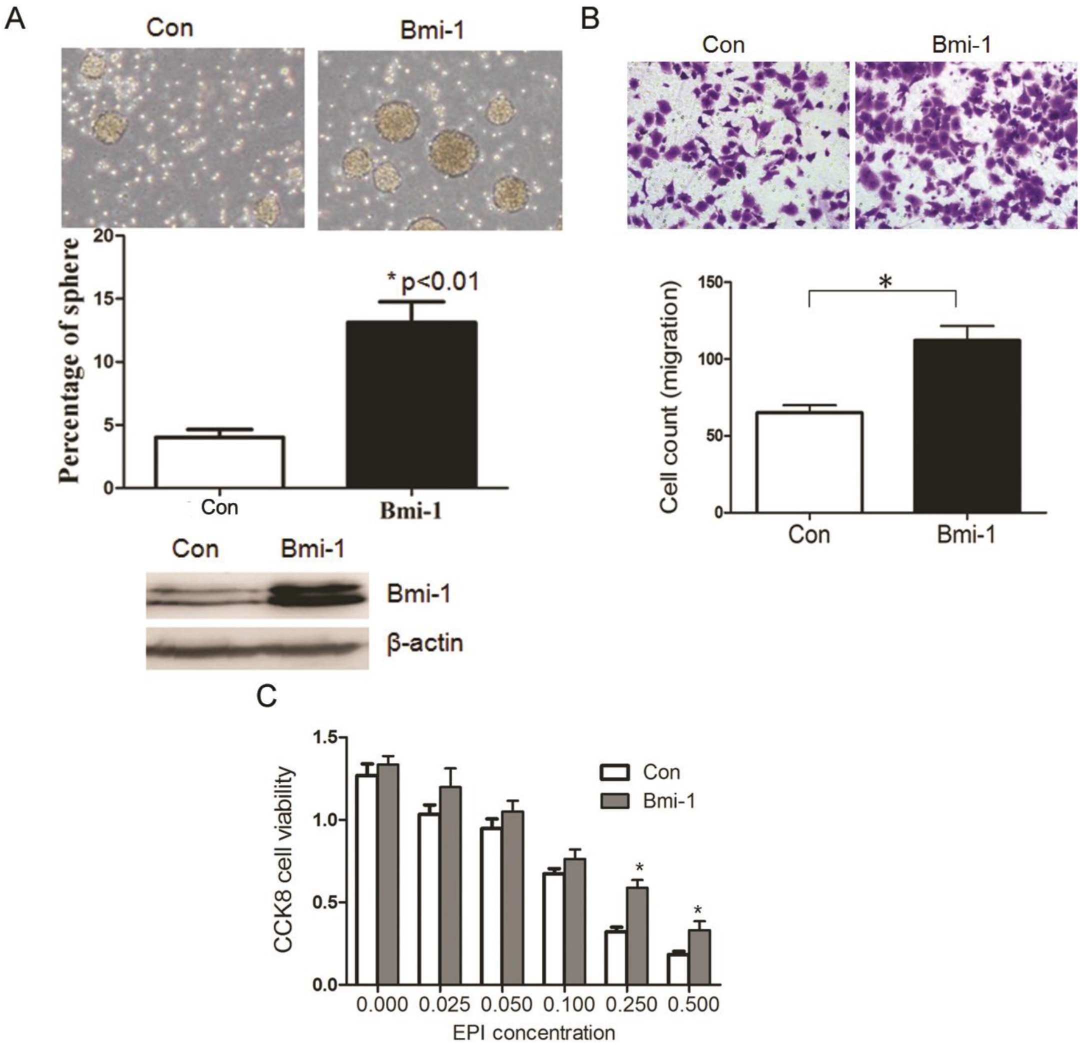

Supplementary Figure S1: Bmi-1 maintains the properties of cancer stem cell-like cells. (**A**) Bmi-1 overexpression increases the size and number of spheres in MKN45 cells. Overexpression of Bmi-1 was tested in gastric cancer cells and measured by Western blot. (**B**) Bmi-1 overexpression in MKN45 increase of gastric cancer migration ability using a Transwell *in vitro* migration model. (**C**) Bmi-1 overexpression confers gastric cancer cells to chemotherapy resistance. Bmi-1 overexpressed cells and control cells was treated by Epirubicin (EPI) for 72 h at different concentrations and detected by CCK8 assay. *$P < .05$.
